# Metabolic Profiling of *Jasminum grandiflorum* L. Flowers and Protective Role against Cisplatin-Induced Nephrotoxicity: Network Pharmacology and In Vivo Validation

**DOI:** 10.3390/metabo12090792

**Published:** 2022-08-25

**Authors:** Moneerah J. Alqahtani, Sally A. Mostafa, Ismail A. Hussein, Seham Elhawary, Fatma A. Mokhtar, Sarah Albogami, Michał Tomczyk, Gaber El-Saber Batiha, Walaa A. Negm

**Affiliations:** 1 Department of Pharmacognosy, College of Pharmacy, King Saud University, P.O. Box 2457, Riyadh 11451, Saudi Arabia; 2 Department of Medical Biochemistry and Molecular Biology, Faculty of Medicine, Mansoura University, Mansoura 35511, Egypt; 3 Department of Pharmacognosy and Medicinal Plants, Faculty of Pharmacy (Boys), Al-Azhar University, Cairo 11884, Egypt; 4 Department of Pharmacognosy, Faculty of Pharmacy, Cairo University, Cairo 11562, Egypt; 5 Department of Pharmacognosy, Faculty of Pharmacy, ALSalam University, Al Gharbiya, Kafr El Zayat 31616, Egypt; 6 Department of Biotechnology, College of Science, Taif University, P.O. Box 11099, Taif 21944, Saudi Arabia; 7 Department of Pharmacognosy, Medical University of Białystok, ul. Mickiewicza 2a, 15-230 Białystok, Poland; 8 Department of Pharmacology and Therapeutics, Faculty of Veterinary Medicine, Damanhour University, Damanhour 22511, Egypt; 9 Department of Pharmacognosy, Faculty of Pharmacy, Tanta University, Tanta 31527, Egypt

**Keywords:** enrichment analysis, LC-MS/MS, MAPK pathway, gene expression, protein–protein interaction, network pharmacology

## Abstract

Cisplatin (CP) is a powerful chemotherapeutic agent; however, its therapeutic use is restricted due to its nephrotoxicity. In this work, we profiled the phytoconstituents of *Jasminum grandiflorum* flower extract (JGF) using LC-MS/MS and explored the possible molecular mechanisms against acute renal failure through pharmacological network analysis. Furthermore, the possible molecular mechanisms of JGF against acute renal failure were verified in an in vivo nephrotoxicity model caused by cisplatin. LC-MS analysis furnished 26 secondary metabolites. Altogether, there were 112 total hit targets for the identified metabolites, among which 55 were potential consensus targets related to nephrotoxicity based on the network pharmacology approach. Upon narrowing the scope to acute renal failure, using the DisGeNET database, only 30 potential targets were determined. The computational pathway analysis illustrated that JGF might inhibit renal failure through PI3K-Akt, MAPK signaling pathway, and EGFR tyrosine kinase inhibitor resistance. This study was confirmed by in vivo experiment in which kidneys were collected for histopathology and gene expression of mitogen-activated protein kinase 4 (MKK4), MKK7, I-CAM 1, IL-6, and TNF receptor-associated factor 2 (TRAF2). The animal-administered cisplatin exhibited a substantial rise in the expression levels of the MMK4, MKK7, I CAM 1, and TRFA2 genes compared to the control group. To summarize, *J. grandiflorum* could be a potential source for new reno-protective agents. Further experiments are needed to confirm the obtained activities and determine the therapeutic dose and time.

## 1. Introduction

Cisplatin is a powerful chemotherapeutic drug that can be used to cure tumors. However, CP has a number of side effects, including nephrotoxicity, which is linked to a high morbidity and mortality rate [1,2]. Nephrotoxicity is caused by cisplatin due to apoptosis and necrosis [3], and tubules inflammation [4]. The oxidative stress triggered by cisplatin leads to the development of renal tubule damage [5]. The formation of reactive oxygen and nitrogen species (ROS and RNS) alters the structure and function of cellular membranes. [6]. Consequently, their buildup in the kidneys and lysosomes [7] elucidated the mechanisms for CP-induced nephropathy [8]. Whereas many pathways for CP-induced nephrotoxicity have been investigated, including mitochondrial malfunction, inflammation, DNA damage, oxidative stress, and apoptosis, the ultimate process is still unknown [9,10].

Therefore, antioxidants and free radical scavengers can aid in the prevention of cisplatin-induced nephrotoxicity. Cisplatin causes apoptosis by disrupting DNA [11]. Many signaling pathways that can be activated by lipid peroxidation and oxidative stress affect cell survival or death in response to cisplatin [12]. Chemical and physical stressors stimulate the mitogen-activated protein kinase (MAPK) pathways, which modulate differentiation, proliferation, and death [13]. The three main MAPK pathways terminate in ERK, p38, and JNK/SAPK enzymes.

Cisplatin is also seen in numerous cell lines as renal epithelial cells, which stimulate these three pathways [14,15]. Inflammation, cell cycle regulation, and differentiation are all affected by p38 MAPK [16]. However, its significance in cancer treatment is unclear. Some researchers believe that p38 MAPK regulates the p53-mediated response to cisplatin [17,18,19,20].

Plants and their components are a better choice for treating ailments than any manufactured chemical [21]. The medicinal plant *Jasminum grandiflorum* Linn. (Family: Oleaceae) is widely utilized for both decorating and therapeutic value. *J. grandiflorum* flower (JGF) is traditionally used to treat mental illness, wounds, infections, skin injuries such as conjunctivitis and dermatitis, dysmenorrhea, spasms, ulcers, and cancer. In China, JGF is also extensively drunk as a heat-clearing and purifying beverage [22,23]. Several beneficial components, including flavonoids, iridoids, triterpenoids, secoiridoids, and lignans, have been identified from *J. grandiflorum* [24,25].

Although other *Jasminum* species and different organs have been investigated, the active metabolites of *J. grandiflorum* flowers (JGF) growing in Egypt have never been studied [26,27]. Here, we analyzed the bioactive compounds of JGF extract using high-performance liquid chromatography coupled with photodiode array detector/mass spectrometry (HPLC-PDA-MS/MS), and then used network pharmacology to explore the connections between the active constituents of JGF, possible protein targets, and hub-signaling pathways related to acute renal failure. Furthermore, in vivo studies investigating JGF’s antioxidant activity in cisplatin-induced renal damage treatment in rat models corroborated the molecular processes predicted by the network pharmacology method against acute renal failure.

## 2. Materials and Methods

### 2.1. JGF Extraction

*Jasminum grandiflorum* Linn. flower powder was made from dried fine crushed flowers collected from Keram fields in El-Behaira Governorate, Egypt. Dr. Mohammed El-Gebaly (Consultant in Orman Garden) verified the plant, and a voucher sample (# 3.10.16.6) was preserved at the Department of Pharmacognosy, Cairo University.

The powdered flower material (1 kg) was extracted with 80% aqueous methanol by percolation (3 × 3 L). The extract was concentrated under reduced pressure to afford a residue (108.8 g) of dry *J. grandiflorum* flower extract (JGF).

### 2.2. JGF Analysis

A Thermo Finnigan Surveyor Plus HPLC apparatus connected to a quaternary pump, a Surveyor UV–Vis photodiode array detector, and an LCQ-Duo ion trap mass spectrometer, via an electrospray ionization source, was utilized. The experiment was performed on a Gemini C18 110 Å (150 × 2 mm, 5 μm). A gradient of water and acetonitrile (ACN) (0.1% formic acid each) was applied from 5% to 30% ACN over 90 min, with a flow rate of 1 mL/min and a 1:1 split before the ESI source

The injection volume was 5 μL with 1 mL/min flow rate. For the negative ionization mode, the following were the ESI conditions: 350 °C, 35 psi nebulizer pressure, 10 L/min N2 drying gas flow rate, mass range 50–2000 m/z. The system was controlled with Xcalibur^TM^ 2.0.7 software. The negative ion mode operating parameters of the MS were employed [28].

### 2.3. Prediction of Bioactive Ingredients of JGF

All the compounds of JGF by LC/MS/MS analysis were considered as candidate compounds. As an online chemical database, PubChem Database (http://pubchem.ncbi.nlm.nih.gov/ accessed on 13 March 2022) was utilized to obtain the chemical data of identified compounds (3D molecular structures, PCIDs, conocial smiles). The 3D molecular structure files were uploaded to the Binding DB database (https://www.bindingdb.org/bind/index.jsp accessed on 20 March 2022), an online tool for identifying drug targets. We obtained anticipated drug targets for each metabolite using this web tool. We identified the target genes with a normalized fit score of >0.7 as prospective JGF targets after combining the duplicate data [29].

### 2.4. Potential Targets Intersection of JGF with Disease

Using the NCBI Gene database (https://www.ncbi.nlm.nih.gov/gene/ accessed on 22 March 2022) [30], and the Online UniProtKB/Swiss-Prot database (https://www.uniprot.org/help/uniprotkb accessed on 25 March 2022) [31], the genes associated with renal fibrosis were gathered from DisGeNet and NCBI databases [32]. The keywords used were “kidney disease”, “acute kidney insufficiency”, “chronic kidney disease”, “chronic kidney failure”, “kidney injury”, “kidney insufficiency”, “acute kidney failure”, kidney ischemia”, prerenal acute kidney”, and “glomerular filtration”. The targets were annotated human genes in this experiment. The interaction of the collected targets was then assigned as a possible therapeutic target. The STRING database was utilized to determine the interaction connection between target proteins.

### 2.5. Protein–Protein Interaction (PPI) Data

The PPI data were acquired from the STRING database (https://string-db.org/cgi/network?taskId=bIDN4htc9NBY&sessionId=bZWvNlZHMn9h accessed on 27 March 2022) [33], as a reliable database for predicting direct and indirect protein–protein interactions. The target proteins were chosen with the human species “Homo sapiens” and a confidence score greater than 0.4. STRING was used to find proteins that interacted with the indicated targets of JGF-isolated compounds directly or indirectly and acute renal failure [34].

### 2.6. Network Construction and Visualization

The Cytoscape network analysis program version 3.9.0 (a software platform that visualizes complex networks and integrates the results) was used to construct compound–target, protein–protein interaction (PPI), and compound–target–disease networks [35]. The difference was deemed significant at *p* < 0.05. Nodes represent targets, compounds, and renal diseases in the graphical network, while edges represent corresponding interactions.

### 2.7. Pathway and Functional Enrichment Analysis

The represented pathways related to JGF and acute renal failure were retrieved by the KEGG (Kyoto Encyclopedia of Genes) enrichment analyses (https://www.genome.jp/kegg accessed on 4 April 2022) [36] and Gene Ontology (ShinyGO) database (http://bioinformatics.sdstate.edu/go/ accessed on 7 April 2022) [37] to investigate the biological processes, cellular components, molecular functions, and involved pathways.

### 2.8. Animals

The experiment was performed under the guidelines of the Research Ethical Committee (Faculty of Pharmacy, Tanta University, Egypt, Approval #PO-21-00109). Male Sprague Dawley (230–260 g) rats were housed under standard conditions of temperature (22 ± 1 °C) and humidity (50–55%), and a 12-h light/dark cycle. There was plenty of food and drink.

### 2.9. In Vivo Experimental Design

The rats were divided into four groups (15 each) at random: the first group was given saline (2.5 mL/kg) intraperitoneally (IP) (normal control group); for the second group, a single dosage of 5 mg/kg CP (Pfizer Company) (1 mg/mL sterile concentrate) was given IP (CP group). For 14 days, the third group was given 40 mg/kg JGF dissolved in water orally (JGF group). The fourth group was given 40 mg/kg JGF dissolved in water orally for two weeks, with a single CP (5 mg/kg, i.p.) dosage on the tenth day. All rats were weighed and anesthetized before decapitation 24 h after the last treatment. Sera were separated after blood samples were collected. The kidneys were removed right away in an ice-cold saline solution. For histopathological and gene expression research, portions of kidneys were sliced into small pieces.

### 2.10. Estimation of Blood Urea Nitrogen and Serum Creatinine

Blood urea nitrogen (BUN) was monitored spectrophotometrically following Tabacco et al. [38]. In summary, serum was diluted 1:4 in normal saline, and 5 μL of diluted serum and standard was added to microplate wells, followed by 150 μL of urease mix solution. At room temperature, the plate was incubated for 15 min with shaking. Each well was then filled with 150 μL of alkaline hypochlorite. After 10 min at room temperature, using a microplate reader, the absorbance of each sample was measured in duplicate at 620 nm. A standard curve was prepared to calculate the concentration. Serum creatinine was detected following the procedures of Fabiny and Ertingshausen [39]. Briefly, after 30 s, picric acid (17.5 mmol/L final concentration)/NaOH solution (0.16 mol/L final concentration) was added to 100 μL of serum samples, and the standard and the absorbance of the standard and sample were measured after 2 min. The creatinine concentration was then estimated by dividing the sample’s delta absorbance by the control’s delta absorbance multiplied by the standard concentration.

### 2.11. Determination of Malondialdehyde (MDA) of Lipid Peroxidation

The concentration of MDA was determined in tissues using the method previously established by Satoh [40] and Ohkawa et al. [41], due to MDA being the primary product of membrane lipid peroxidation. The idea of this approach is based on the development of a pink color as a result of MDA and thiobarbituric acid reacting. The reaction yields a pink thiobarbituric acid reactive substance (TBARS) with a spectrophotometric wavelength of 532 nm.

### 2.12. Determination of Glutathione Levels (GSH) in Kidney Tissues

GSH concentration was measured using Beutler’s method [42]. The procedure is established by reducing 5,5' dithiobis (2—nitrobenzoic acid) with GSH to generate a yellow compound. The reduced chromogen’s absorbance could be determined at 405 nm and was directly proportional to GSH content.

### 2.13. RNA Extraction and Gene Expression Studies

TRIzol reagent (Invitrogen, Waltham, MA, USA) was used to purify the RNA. Thermo Scientific Maxima First Strand DNA Synthesis Kit with dsDNase was then used to make complementary DNA (Thermo Fisher Scientific, Rockford, IL, USA). The control gene was the B actin gene. Primer 3 PLUS software was used to create gene primer sets (v. 0.4.0; 163 https://frodo.wi.mit.edu/; accessed on 29 December 2021 Table S1). Real-time PCR assays were conducted using the Applied Biosystem 7500 real-time PCR detection system (Life Technologies, Carlsbad, CA, USA) with the SensiFAST SYBR Lo-ROX PCR Master Mix Kit (Bioline, Toronto, UK). The overall reaction volume was 20 μL, with the following thermal reaction profile: initial denaturation at 95 °C for 2 min followed by 40 cycles of 95 °C for 5 s, then 60 °C for 30 s. The method (2^−ΔΔCt^) was used for the calculation of fold induction values [43].

### 2.14. Histopathology Assessment

Each group’s kidneys were preserved in a 10% neutral buffered formaldehyde solution. The conventional process was followed for tissue dehydration, xylene cleaning, and paraffin embedding. Hematoxylin and eosin, as well as periodic acid schief (PAS), were used to stain sections cut using a rotary microtome at 5–7 μm thickness.

### 2.15. Statistical Analysis

All the tests were done in triplicate, and the data were reported using SPSS software version 26 as means and standard deviation (SD) (IBM Corp., Version 26.0. Armonk, NY, USA). ANOVA was used to compare the parameters obtained from the various tested groups. The statistical significance of the received data was assessed using *p* < 0.05.

## 3. Results

### 3.1. JGF Phytochemical Profile

A total of 26 secondary metabolites were tentatively identified in JGF using LC-MS/MS. The main compounds are divided into several subclasses, including secoiridoids, flavonoids, and phenolic and organic acids. Comprehensive profiling is presented in Table 1, and the total ion chromatogram (TIC) of JGF is displayed in Supplementary Figure S1.

### 3.2. Compound–Target Network Construction

**Figure 1:**
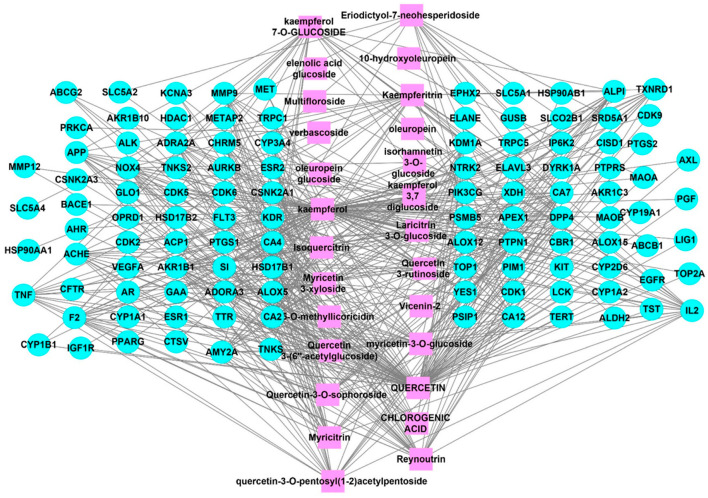
Compound–common targets of the identified compounds of *J. grandiflorum* flower (JGF). Pink rectangle shapes represent the identified compounds from JGF; blue circles indicate common targets of identified compounds. Edges indicate interactions between identified compounds and common targets.

A total of 112 targets were found among the 26 identified chemicals in the NCBI Gene and UniProtKB/Swiss-Prot databases. A total of 55 potential renal disease targets were identified from the DisGeNet database, among which only 30 targets are related to acute renal failure. Taking the intersection of the 112 potential targets and the compounds in JGF into account, we constructed a compound–target network using Cytoscape (Figure 1 and Table S2), which included 138 nodes (26 for potential phytoconstituents and 112 for protein targets) with 547 edges. The top hit targets for the identified compounds were PTPN1, NOX4, and TNF, with no edges > 10.

**Figure 2:**
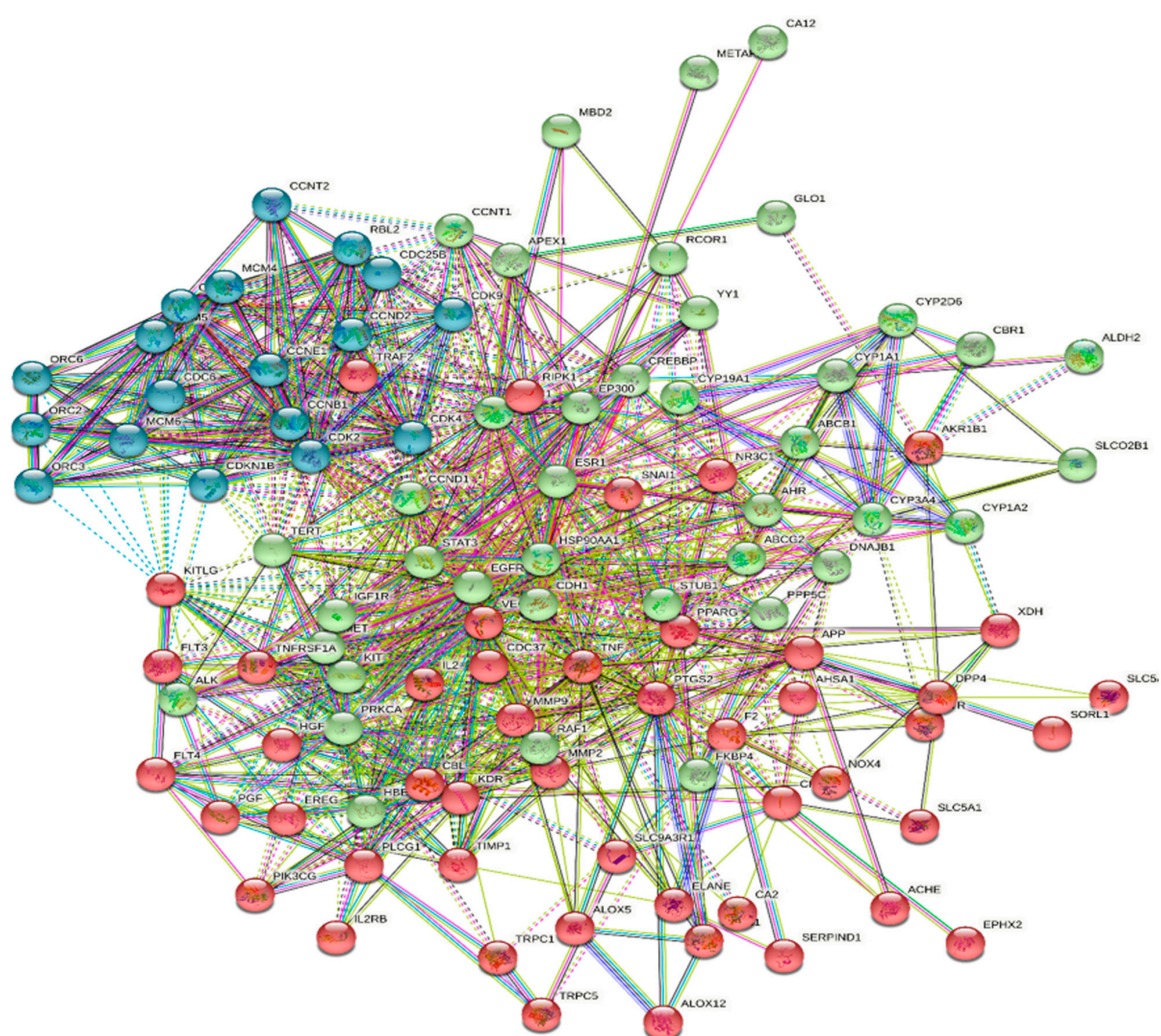
PPI of the hit targets of the identified compounds of *J. grandiflorum* flower (JGF): results represented in four clusters obtained from the STRING online database; dotted lines represent interactions between clusters, solid lines represent interactions between proteins.

The 112 possible therapeutic target genes were loaded into the STRING database, which supplied predicted interaction data, and then imported into Cytoscape 3.9.0 for analysis and network construction (Figure 2). In the PPI network, the top 17 genes, prioritized by interactions, were gathered to be the hub targets, each represented by edges > 25, namely HSP90AA1, TNF, ESR1, VEGFA, EGFR, PPARG, PTGS2, HSP90AB1, CA4, MMP9, AR, KDR, CYP3A4, HDAC1, APP, CDK1, and CDK2. These genes contributed to the gene–disease interactions. The PPI was represented in 4 clusters, with 131 nodes, 831 edges, and an average local clustering coefficient equal to 0.532.

### 3.3. Potential Targets Intersection of JGF with Disease

**Figure 3:**
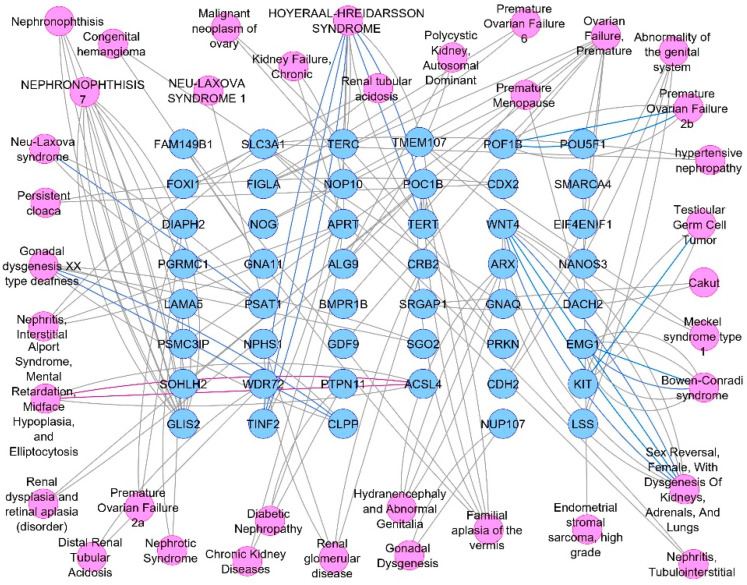
Targets—urogenital diseases network analysis. Blue circles represent targets, and pink ones represent renal disease types.

**Figure 4:**
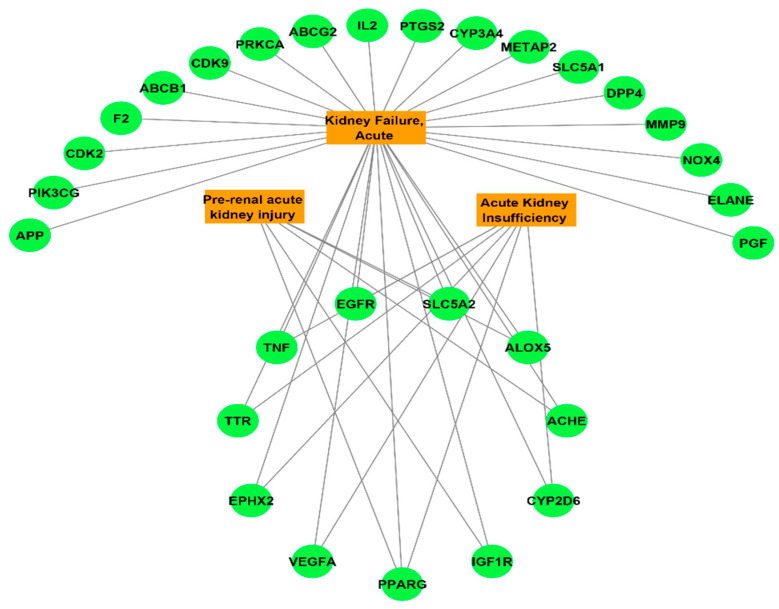
Targets—acute renal failure network analysis. Green circles represent the target, and orange rectangles represent types of renal failure.

The DisGeNET online database was used to interpret the associated diseases with the identified 112 target hits of the JGF-identified compounds. Upon filtration of the results to narrow the scope to urogenital disorders, a total of 47 genes were obtained (Figure 3). To direct the research scope to acute renal failure disorders, 30 genes were detected (Figure 4).

### 3.4. Compounds–Common Targets–Renal Failure Pharmacology Network

**Figure 5:**
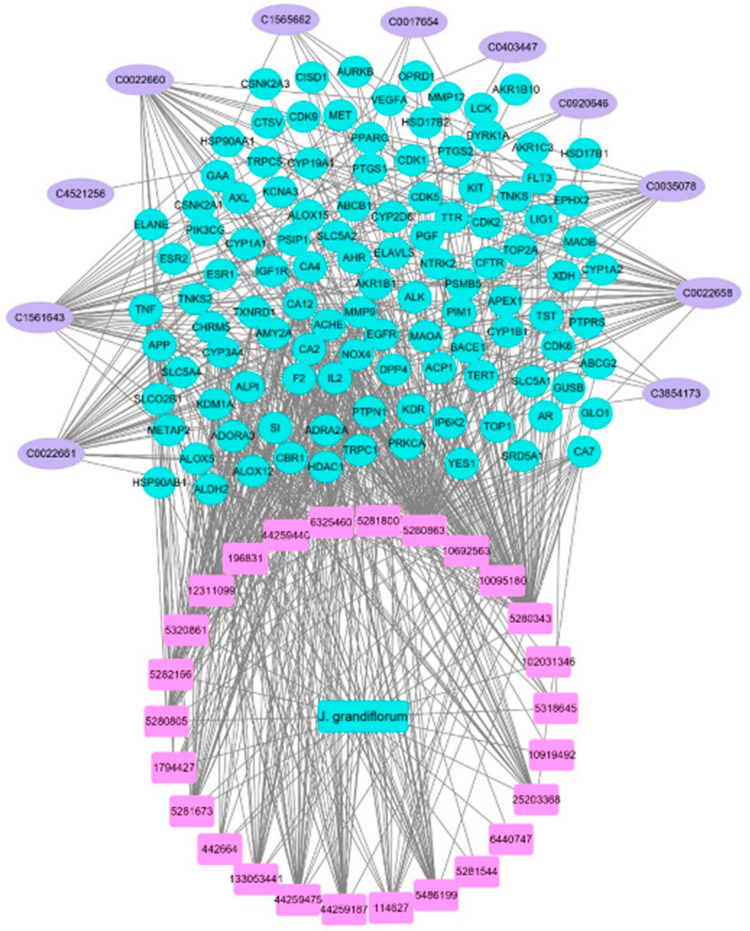
The total merged networking of *J. grandiflorum* identified compounds, targeted genes, and targeted renal diseases in code forms. The pink oval shapes represent renal diseases, pink rectangles represent identified compounds (PCIDs), blue circles represent target genes (gene IDs), and the blue rectangle represents JGF.

The combination and merging of the JGF–compounds, compounds–targets, and targets–non-cancer renal diseases networks formed the complete pharmacology network that correlates the identified compounds from JGF, as described in Figure 5.

### 3.5. Target Genes–Pathways Network

**Figure 6:**
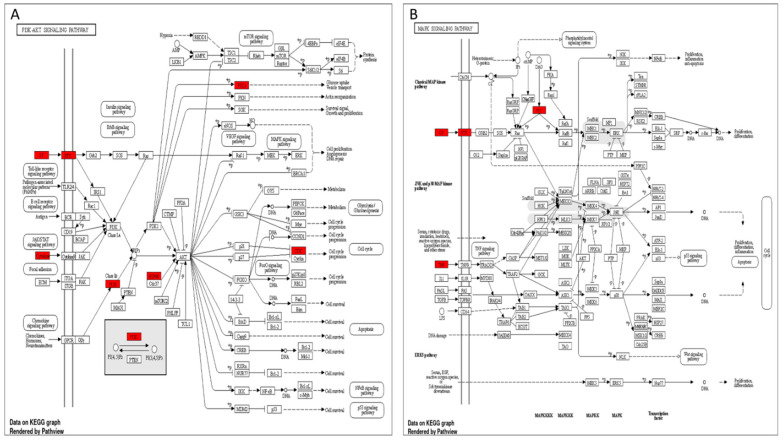
The top pathways enriched to the target hits of JGF involved in the acute renal failure diseases illustrate the directly involved genes among JGF target hits. Red rectangles are the target genes in each pathway: (**A**) PI3K-Akt signaling pathway, (**B**) MAPK signaling pathway.

To investigate the probable pathways of JGF on kidney disorders, the pathway enrichment of 112 probable targets was targeted by 26 identified compounds interacting with renal diseases using the KEGG and ShinyGO databases. The pathways analysis conducted with the KEGG diagram illustrated the top 24 pathways annotated by the target genes. Upon focusing on the pathways involved in acute renal failure disorders, three signaling pathways were identified: PI3K-Akt, MAPK, and EGFR tyrosine kinase inhibitor resistance. Each pathway diagram is illustrated to elaborate the specific genes among our targets involved directly in the nominated pathways as described by the KEGG labeled diagrams (Figure 6 and Table S3).

### 3.6. GO Enrichment Analysis

**Figure 7:**
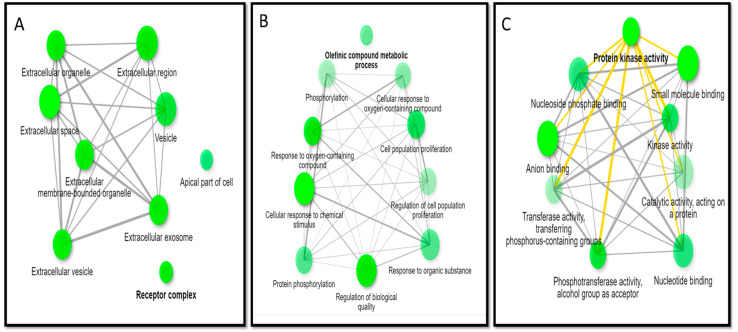
Gene ontology analysis of all target genes of active compounds of JGF: (**A**) cellular components, (**B**) biological processes, (**C**) molecular functions.

Using GO terms, the gene ontology results are illustrated as networks (Figure 7), representing the analysis of the cellular components and illustrating the role of the target hits of JGF phytometabolites. The major identified cellular components according to fold enrichment are receptor complex (GO:0043235), apical part of cell (GO:0045177), and perinuclear region of cytoplasm (GO:0048471) (Table S4). The molecular functions enriched to all targets were arranged according to fold enrichment, and the top molecular functions are protein kinase activity (GO:0004672), phosphotransferase activity, alcohol group as acceptor (GO:0016773), and oxidoreductase activity (GO:0016491) (Table S5). The biological processes enriched for all the target hits of JGF were arranged according to fold enrichment, and the top biological processes are olefinic compound metabolic process (GO:0120254), cellular response to oxygen-containing compound (GO:1901701), and response to oxygen-containing compound (GO:1901700) (Table S6).

### 3.7. In Vivo Studies

#### 3.7.1. Effects of JGF on Bodyweight, BUN, and Serum Creatinine

**Figure 8:**
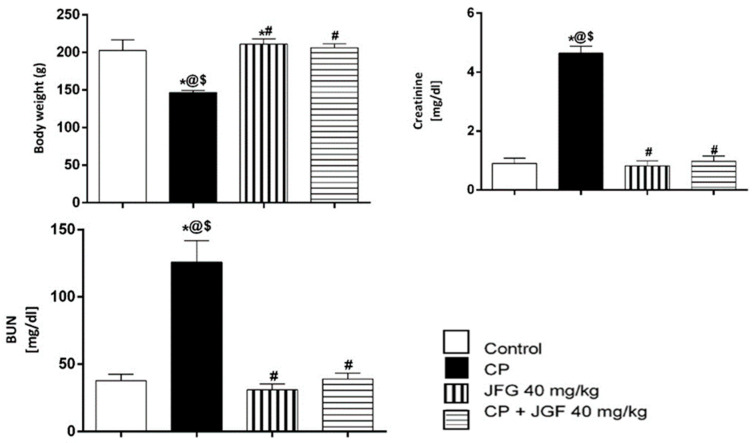
Effects of *J. grandiflorum* (JGF) on body weight, renal BUN, and serum creatinine. *, #, @, $ indicates significant change from control, cisplatin group (CP), *J. grandiflorum*-treated group (JGF), and cisplatin + *J. grandiflorum*-treated group (CP + JGF), respectively.

The impact of CP, JGF, and their combination on rat body weight is shown in Figure 8. Compared to the control group, CP-treated animals lost weight significantly (*p* < 0.05) at the end of the experiment. Interestingly, JGF treatment alone increased the body weight compared to the control and cisplatin groups. In addition, treatment of JGF in combination with CP attenuated the deleterious effects of CP and increased the declined body weight (*p* < 0.05, Figure 8).

Cisplatin elevated BUN and serum creatinine levels substantially compared to the control group (*p* < 0.001). However, JGF combined with CP completely reversed the elevated levels, which returned to normal levels in the control group (Figure 8). Of note, there were no substantial differences in BUN and serum creatinine in the JGF group compared to the control group.

#### 3.7.2. Effects of JGF on Renal Oxidative Stress

**Figure 9:**
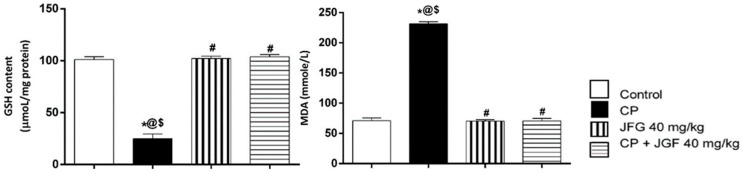
Effects of *J. grandiflorum* (JGF) on renal oxidative stress (GSH and MDA). *, #, @, $ indicates significant change from control, cisplatin group (CP), *J. grandiflorum*-treated group (JGF), and cisplatin + *J. grandiflorum*-treated group (CP + JGF), respectively, at *p* < 0.05.

CP elevated MDA and lowered GSH levels in renal tissue substantially compared to the control group. Remarkably, pretreatments with JGF inverted the adverse effects and returned them to normal levels, much like in the control group (Figure 9). Compared to the control group, treatment of JGF alone did not affect MDA or GSH levels.

#### 3.7.3. Effects of JGF on the Levels of Gene Expression

**Figure 10:**
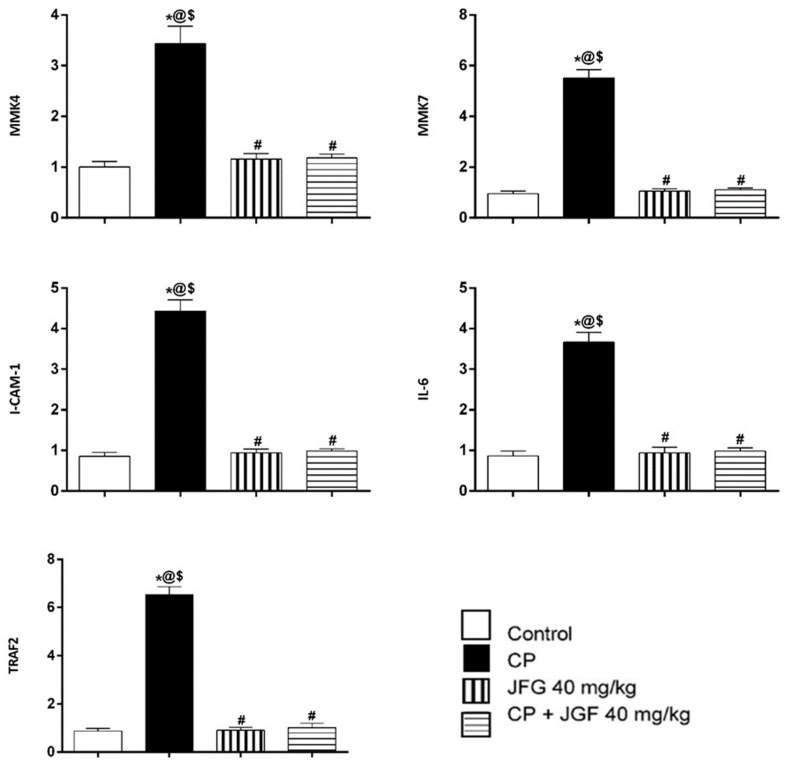
Effects of *J. grandiflorum* (JGF) on the MMK4, MMK7, ICAM-1, IL-6, and TRAF2 gene expression levels. *, #, @, $ indicates significant change from control, cisplatin group (CP), *J. grandiflorum*-treated group (JGF), and cisplatin + *J. grandiflorum*-treated group (CP + JGF), respectively, *p* < 0.001.

Figure 10 depicts the effects of CP, JGF, and their combination on MKK4, MKK7, I-CAM-1, IL-6, and TRAF 2 expression levels in kidney tissues. CP alone resulted in a remarkable rise in tested markers (*p* < 0.001) compared to the control group. Pretreatments with JGF inverted the elevated levels, leading to a complete setback to normal values (Figure 10). In the JGF group, no significant alterations in any of the tested markers were observed.

#### 3.7.4. Histopathology

**Figure 11:**
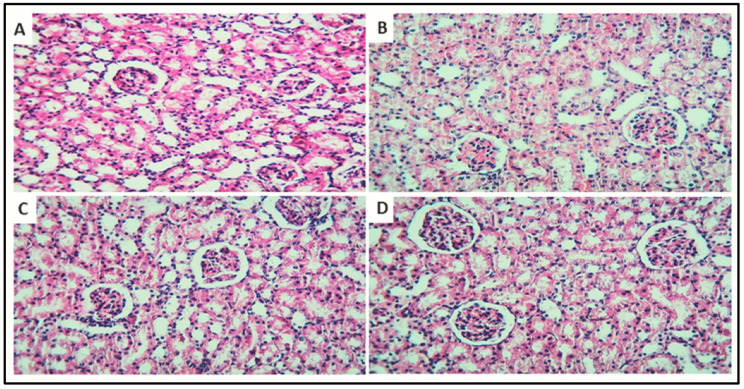
Effects of JGF on histopathological examination of rat kidney. (**A**) The section in the cortex from the normal group consists of glomeruli, proximal convoluted tubules, and distal convoluted tubules. (**B**) The section in the cortex of a rat that received cisplatin shows severe effects on the renal tubules, with signs of tubular cell lining vacuolation and intratubular cast deposition (arrowheads). (**C**) A section in the cortex of a rat that received JGF, consisting of glomeruli, proximal convoluted tubules, and distal convoluted tubules. (**D**) The section in the cortex of a rat that received cisplatin + JGF shows almost normal renal glomeruli and tubules, apart from minimal tubular cell lining vacuolations ((H,E) section × 200 bar = 50 µm).

Figure 11 depicts the impact of CP and JGF on histological alterations in renal tissues. Under a light microscope, the kidney tissues from the normal and treated rat groups were examined. The kidneys of control rats showed no glomeruli histological abnormalities, proximal convoluted tubules, or distal convoluted tubules. The section in the cortex of rats that received cisplatin showed severe effects on the renal tubules, with signs of tubular cell lining vacuolation and intratubular cast deposition. The section in the cortex of rats that received JGF showed normal architecture. The section in the cortex of rats that received cisplatin + JGF showed almost normal renal glomeruli and tubules, apart from minimal tubular cell lining vacuolations.

## 4. Discussion

CP is an anticancer medicine that was established to treat a variety of malignancies, including testicular, head, neck, ovary, lung, and breast cancers [1]. However, its dose-limiting adverse effect is nephrotoxicity [44]. Acute renal damage was also detected in roughly 20–30% of patients taking CP [45], and hypomagnesemia in approximately 40–100% of patients [46], as well as distal renal tubular acidosis, hypocalcemia, renal salt wasting, hyperuricemia, and Fanconi-like syndrome [47].

Nephrotoxicity is defined as a decrease in renal function that results in an elevation in serum creatinine and blood urea levels as a result of CP [48]. Serum creatinine and BUN levels were considerably higher in CP-treated rats compared to the untreated group in this study, indicating that CP caused nephrotoxicity as indicated by a reduction in glomerular filtration rate. JGF significantly reduced the increased serum creatinine and BUN levels caused by CP, restoring them to normal values in the control group.

Other research suggests that CP causes ROS and an immunological response, both of which are nephrotoxic mediators [49,50]. MDA and GSH were assessed as indicators for oxidative stress in this investigation. The action of cisplatin on renal tissue resulted in a considerable rise in MDA and a reduction in GSH. The administration of JGF, on the other hand, resulted in considerable reductions in lipid peroxidation and stimulated a rise in GSH content in the kidney. As a result of the improved oxidant status, JGF can protect the kidney from damage caused by cisplatin. JGF may furnish its nephroprotective activity through its antioxidant properties.

The p38-MAPK stress pathway, activated by inflammatory cytokines such as TNF-α or IL-1, is a critical regulator of cell apoptosis [51]. After cisplatin injury, the amount of inflammatory cytokines and chemokines is raised in the kidney [52]. CP boosted the expression of inflammatory cytokines such as IL-6 in the current study. TNF-α, IL-1β, macrophage inflammatory protein-2 (MIP-2), monocyte chemoattractant protein-1 (MCP-1), ICAM-1, and TGF-β were all upregulated in the kidneys of CP-treated animals [53]. JGF administration enhanced the CP-induced rise in IL-6 expression levels.

Due to the highly complex biochemistry of plants, which includes a variety of semi-polar molecules, including important secondary metabolite groups, which may be best separated and detected by LC-MS techniques, LC-MS-based approaches are anticipated to be particularly significant for plants. Mass spectrometry is a reliable detection method, and using tandem mass spectrometry can improve selectivity and specificity. The analysis of several phytochemicals can be conducted using LC-MS since it is sensitive and selective [54].

The negative ionization modes of LC-MS/MS analysis of *J. grandiflorum* flower extract resulted in the detection of twenty-six bioactive metabolites compounds. The identified metabolites belong to several phytochemical classes, such as secoiridoids, phenolic acids, and flavonoids, in agreement with the previous literature [27,55].

HPLC–PDA–MS profiling of JGF produces extremely rich metabolite profiles. LC/MS analysis of JGF demonstrated that it has significant flavonoids and polyphenolic content. The different flavonoid subclasses (flavone, flavanone, and flavonols) were also represented by aglycones and glycosides.

Flavonoids are essential in a wide range of nutraceutical, pharmacological, medical, and cosmetic uses because they are linked to a wide range of health-promoting effects [56]. This is because they possess strong anti-oxidative, anti-inflammatory, anti-mutagenic, antimicrobial, anti-carcinogenic, vascular, and other therapeutic properties, as well as the ability to modify crucial cellular enzyme processes [57]. The present study agreed with previous reports that demonstrate the protective effect of natural products containing flavonoids as antioxidants and anti-inflammatory agents in kidney injury [58,59,60].

Cisplatin-induced inflammatory cytokines primarily rely on forming reactive oxygen species (ROS), NFκB activation, and p38 MAPK activation. TNF-α and IL-1 substantially activate the stress-activated group of MAPKs (JNK and p38) [52]. This was supported by the current study, which found that a single dose of CP increased JNK and P38 expression. TNF activates JNK through the TNF receptor-associated factor (TRAF) class of adaptor proteins [61].

The present study suggests that cisplatin induced TRAF2 overexpression is the cause of nephrotoxicity and apoptosis. The reduction in TRAF2 expression in kidney tissues after JGF administration in CP-treated rats implies that JGF may safeguard against nephrotoxicity caused by CP through regulating apoptotic pathways. The TRAF2 adapter protein is recruited when TNF receptors are activated [62,63]. TRAF2 expression needs to be activated for TNF to activate JNK [62].

According to a previous study, JNK genes have an essential role in modifying the pro- and anti-apoptotic proteins in the mitochondria in the nephrotoxicity caused by chemotherapy [64]. By blocking anti-apoptotic proteins, JNK with ROS can increase apoptosis [65]. JNK can also be activated by MKK4 and MKK7 phosphorylation at threonine and tyrosine. MAPKKK phosphorylates two locations in the T-loop to activate MKK4 and MKK7 protein kinases [66].

The MKK7 is mainly triggered by cytokines, whereas MKK4 is activated primarily by environmental stress [67]. In the present work, CP-induced changes in MKK4 and MKK7 expression were reduced in CP-treated rats by supplementing them with JGF. MKK3, MKK4, and MKK6 all activate P38 MAPK [68]. After a single cisplatin dosage, P38 expression was increased in this investigation. Inhibition of p38 MAPK, ERK, or JNK with particular pharmacologic or genetic inhibitors decreased inflammation and kidney injury in multiple investigations. In CP-treated rats, JGF treatment restored P38 expression and decreased apoptosis.

JGF showed a significant nephroprotective effect in rats, and its nephroprotection mechanism could be attributable to the presence of a variety of phytochemicals, including secoiridoids, flavonoids, and phenolic and organic acids with antioxidant properties and free-radical-scavenging properties. Therefore, this plant could be useful in treating renal failure caused by nephrotoxic drugs.

## 5. Conclusions

The metabolites in *J. grandiflorum* were detected using HPLC-PDA-MS/MS, including quercetin, myricetin, kaempferol, isorhamnetin flavonoids, and other secoiridoid derivatives. Our findings could contribute to the understanding of the molecular mechanisms of JGF in CP nephrotoxicity, which were confirmed by in vivo research that supported the molecular processes predicted by the network pharmacology method. Compared to the control group, a single dose of CP to rats caused nephrotoxicity, which was related to a considerable rise in BUN and serum creatinine and a significant increase in MDA in renal tissues. The expression levels of the MMK4, MKK7, I CAM 1, and TRFA2 genes were considerably higher in the cisplatin-treated animals than in the control. Furthermore, the histological investigation revealed that cisplatin induced significant tubular cell lining vacuolation and intratubular cast deposition in the renal tubules. JFG treatment reduced cisplatin-induced gene expression changes and kidney structural and functional abnormalities. Gene expression data were also corroborated by histological investigation of kidney tissues. Our study suggests that the anti-inflammatory effects of JGF can protect from CP-induced nephrotoxicity through lowering oxidative stress and repairing the histopathological changes against cisplatin use. However, further studies should be carried out in the future to reveal the clinical effectiveness of *J. grandiflorum* as a renal protective drug in preclinical and clinical trials.

## Figures and Tables

**Table 1:** Phytochemical profiling of *J. grandiflorum* flower (JGF) in negative mode HPLC-PDA-MS/MS.

No	Compound	RT	*m/z*	Fragments	Ontology
1	1-Caffeoylquinic acid	17.04	353.16	191, 179	Phenolic acid
2	Verbascoside	25.02	623.59	461, 161	Phenylpropanoid
3	Elenolic acid glucopyranoside	27.38	403.95	241, 179	Secoiridoids
4	Quercetin-3-*O*-pentosyl (1–2) acetylpentoside	27.99	607.11	433, 301	Flavonoid glycosides
5	Quercetin 3-rutinoside	30.09	609.22	463, 301	Flavonol glycosides
6	Quercetin-3-*O*-sophoroside	33.14	624.94	463, 301	Flavonol glycosides
7	Myricetin-3-*O*- glucopyranoside	34.10	479.08	317, 195	Flavonoid-3-glycosides
8	Eriodictyol-7-*O*-neohesperidoside	34.14	595.23	289, 163	Flavonoid-7-glycosides
9	Kaempferol 3,7 Di-glucoside	35.13	609.32	285, 179	Flavonoid glycosides
10	10-hydroxyoleuropein	36.21	555.08	539, 249	Secoiridoids
11	Vicenin-2	37.04	593.28	503, 353	Flavonoid 8-*C*-glycosides
12	Oleuropein glucoside	38.75	701.31	529, 223	Secoiridoids
13	Kaempferol-7-*O*-glucoside	40.46	447.34	285, 177	Flavonol glycosides
14	Isorhamnetin-3-*O*- glucoside	41.84	477.03	315, 193	Flavonoid 8-*C*-glycosides
15	Isoquercitrin	43.41	463.08	301, 179	Flavonoid 8-*C*-glycosides
16	Multifloroside	44.83	677.31	661, 539	Secoiridoids
17	Oleuropein	46.46	539.17	377, 233	Secoiridoids
18	Kaempferitrin	47.44	577.31	285, 179	Flavonoid 8-*C*-glycosides
19	Reynoutrin	49.18	433.10	301	Flavonoid glycosides
20	Quercetin 3-(6″-acetylglucoside)	51.08	505.02	301, 271	Flavonol glycosides
21	Myricetin 3-xyloside	56.41	449.05	317, 179	Flavonoid-3-glycosides
22	Laricitrin 3-*O*-glucoside	58.48	493.10	331, 151	Flavonoid glycosides
23	Myricitrin	59.39	463.28	317, 179	Flavonoid-3-*O*-glycosides
24	Kaempferol	61.25	285.30	269, 241	Flavonoid glycosides
25	Quercetin	61.62	301.17	245, 179	Flavonoid
26	5-*O*-Methyllicoricidin	66.82	483.61	203, 177	Flavan

## Data Availability

Data is contained within the article and supplementary material.

## Supplementary Materials

The following supporting information can be downloaded at: https://www.mdpi.com/article/10.3390/metabo12090792/s1, Figure S1. The total ion chromatogram (TIC) of *J. grandiflorum* flower extract (JGF), Table S1. Sequence genes, Table S2. Network analysis of the identified compounds arranged in descending order according to the degree of centrality, Table S3. Top KEGG pathways to the identified target genes corresponding to the identified compounds of Jasminum grandiflorum flowers, number of genes involved in each pathway > 3 genes, Table S4. Top cellular components enriched to the target genes identified as targets to identified compounds from JGF, Table S5. Top Molecular functions enriched to the target genes identified as targets of identified compounds from JGF, Table S6. Top biological process enriched to the target genes identified as targets of identified compounds from JGF.

## Abbreviations

PTPN1	Receptor-interacting serine/threonine-protein kinase 1
NOX4	NADPH oxidase 4
TNF	Tumor necrosis factor
p38 MAPK	Mitogen-activated protein kinase signaling pathway
HSP90AA1	Heat shock protein HSP 90-alpha
ESR1	Estrogen receptor
VEGFA	Vascular endothelial growth factor A
EGFR	Epidermal growth factor receptor
PPARG	Peroxisome proliferator-activated receptor gamma
PTGS2	Cyclooxygenase
HSP90AB1	Heat shock protein HSP 90-beta
CA4	Carbonic anhydrase 4
MMP9	Matrix metalloproteinase 9
AR	Androgen Receptor
CYP3A4	Cytochrome P450 3A4
HDAC1	Histone deacetylase
APP	beta-Glucuronidase (β-glucuronidase)
CDK1	Cyclin-dependent kinase 1
CDK2	Cyclin-dependent kinase 2
KDR	Vascular endothelial growth factor receptor 2
ERK	Extracellular signal-regulated kinases
JNK	c-Jun N-terminal protein kinases (JNK), also known as stress-activated protein kinases
STRING	The STRING database is one of several online resources dedicated to organism-wide protein association networks
DisGeNET	A discovery platform containing one of the largest publicly available collections of genes and variants associated with human diseases
HDAC1	Histone deacetylase
GO	Gene ontology
JGF	*Jasminum grandiflorum* flowers
HPLC-PDA/MS	High-performance liquid chromatographic coupled with diode array detector/mass spectrometry
