# Supplementary material for: Metabolic Profiling of Jasminum grandiflorum L. Flowers and Protective Role against Cisplatin-Induced Nephrotoxicity: Network Pharmacology and In Vivo Validation
Source: Metabolites. 2022 Aug 25;12(9):792. doi: 10.3390/metabo12090792 (PMC9502427; doi:10.3390/metabo12090792)
Supplement: Supplementary file 1 [file metabolites-12-00792-s001.zip › metabolites-1861935-supplementary.pdf]

Article

Metabolic Profiling of *Jasminum grandiflorum* L. Flowers and Protective Role against Cisplatin-Induced Nephrotoxicity: Network Pharmacology and In Vivo Validation

Moneerah J. Alqahtani [1], Sally A. Mostafa [2], Ismail A. Hussein [3], Seham Elhawary [4], Fatma A. Mokhtar [5], Sarah Albogami [6], Michał Tomczyk [7], Gaber El-Saber Batiha [8] and Walaa A. Negm [9,*]

[1] Department of Pharmacognosy, College of Pharmacy, King Saud University, P.O. Box 2457, Riyadh 11451, Saudi Arabia
[2] Department of Medical Biochemistry and Molecular Biology, Faculty of Medicine, Mansoura University, Mansoura 35511, Egypt
[3] Department of Pharmacognosy and Medicinal Plants, Faculty of Pharmacy (Boys), Al-Azhar University, Cairo 11884, Egypt
[4] Department of Pharmacognosy, Faculty of Pharmacy, Cairo University, Cairo 11562, Egypt
[5] Department of Pharmacognosy, Faculty of Pharmacy, ALSalam University, Al Gharbiya, Kafr El Zayat 31616, Egypt
[6] Department of Biotechnology, College of Science, Taif University, P.O. Box 11099, Taif 21944, Saudi Arabia
[7] Department of Pharmacognosy, Medical University of Białystok, ul. Mickiewicza 2a, 15-230 Białystok, Poland
[8] Department of Pharmacology and Therapeutics, Faculty of Veterinary Medicine, Damanhour University, Damanhour 22511, Egypt
[9] Department of Pharmacognosy, Faculty of Pharmacy, Tanta University, Tanta 31527, Egypt
* Correspondence: walaa.negm@pharm.tanta.edu.eg

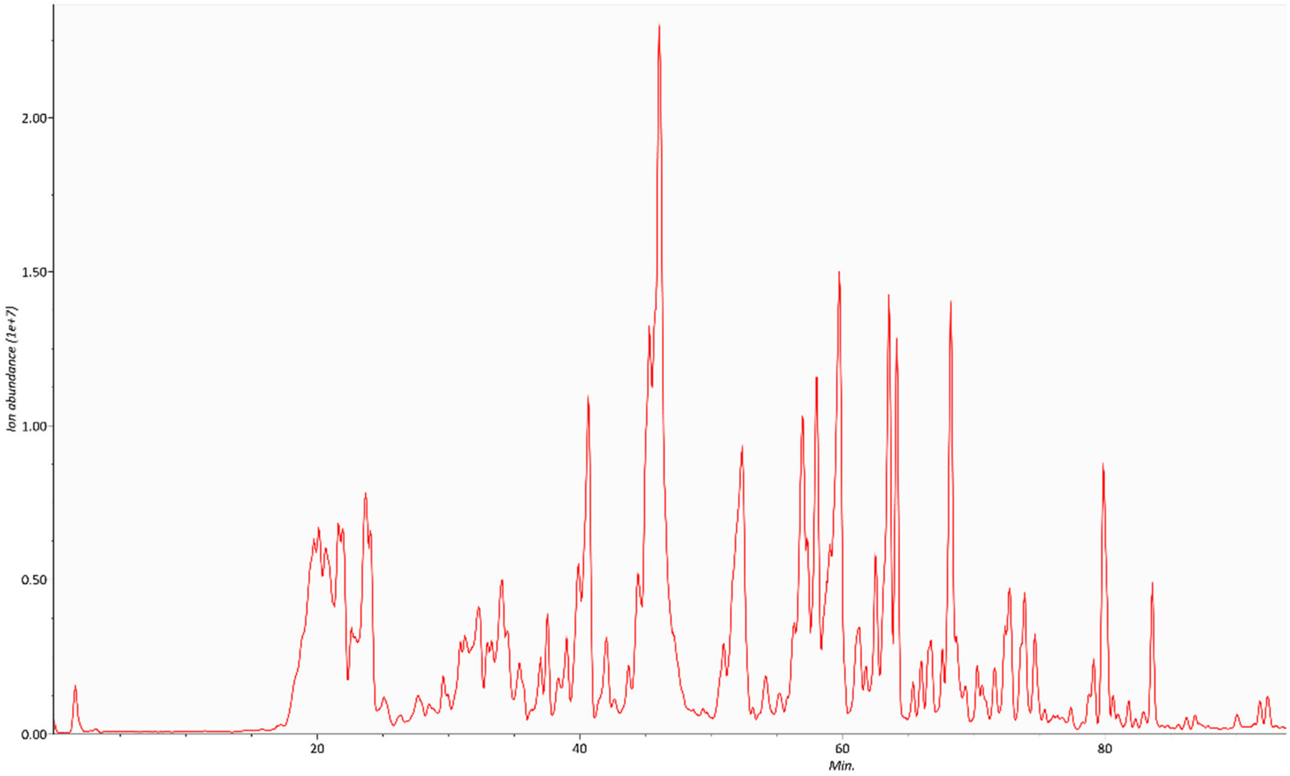

Figure S1. The total ion chromatogram (TIC) of *J. grandiflorum* flower extract (JGF)

Table S1. Sequence genes

Gene	Forward primer	Reverse primer	Product length	Reference sequence
MMK 4	AACTGCTTCTTGGAGCTTTGG	CGGGGTATGACTGCAGAAAATC	184	NM_001316367.1
MMK 7	AACATCCTGCTAGATGAGCGG	TGGGATCTGGAGGGTCGAT	142	NM_001042557.2
I-CAM 1	TGGAGACGCAGAGGACCTTA	AAGAACCACCTTCGACCCAC	131	NM_010493.3
IL-6	TCTGTAGCTCATTCTGCTCTGG	AGTCTCCTCTCCGGACTTGT	162	NM_031168.2
TRAF 2	TGCTGAGTGGTGTTCATCTGG	GCAGACTCACAATGGAGTGGT	137	NM_009422.3
B actin	TGGCTCCTAGCACCATGAAG	AACGCAGCTCAGTAACAGTCC	193	NM_007393.5

Table S2. network analysis of the identified compounds arranged in descending order according to the degree of centrality

Compound name	Degree of centrality	Average Shortest Path Length	Betweenness Centrality	Number Of Undirected Edges
kaempferol	86	1.751824818	0.364133565	86
Quercetin	84	1.737226277	0.382968615	84
Reynoutrin	28	2.467153285	0.025448774	28
Myricetin 3-xyloside	28	2.467153285	0.025448774	28
kaempferol 7-O-GLUCOSIDE	26	2.496350365	0.034676009	26
Laricitrin 3-O-glucoside	26	2.496350365	0.019346305	26
Myricitrin	25	2.510948905	0.017029552	25
Kaempferitrin	25	2.510948905	0.017029552	25
kaempferol 3,7 diglucoside	25	2.510948905	0.017029552	25
myricetin-3-O-glucoside	25	2.510948905	0.017029552	25
Isoquercitrin	25	2.510948905	0.017029552	25
Quercetin 3-rutinoside	24	2.525547445	0.015622781	24
Quercetin-3-O-sophoroside	24	2.525547445	0.015622781	24
Quercetin 3-(6"-acetylglucoside)	24	2.525547445	0.015821347	24
quercetin-3-O-pentosyl (1-2)acetylpentoside	21	2.569343066	0.011551375	21
Eriodictyol-7-neohesperidoside	17	2.686131387	0.110058158	17
Chlorogenic acid	9	2.817518248	0.115658673	9
isorhamnetin 3-O-glucoside	8	2.97810219	0.001040306	8
5-O-methyllicoricidin	3	3.03649635	3.81E-04	3
verbascoside	2	4.759124088	0.01459854	2
Oleuropein	1	4.175182482	0	1
10-hydroxyoleuropein	1	4.175182482	0	1
elenolic acid glucoside	1	4.175182482	0	1
Multifloroside	1	4.175182482	0	1
oleuropein glucoside	1	4.175182482	0	1

Table S3. Top KEGG pathways to the identified target genes corresponding to the identified compounds of Jasminum grandiflorum flowers, no of genes involved in each pathway > 3 genes

No	Pathway	No of Genes	Genes
1	Pathways in cancer	11	CDK2 EGFR F2 IGF1R IL2 MMP9 PGF PPARG PRKCA PTGS2 VEGFA
2	* PI3K-Akt signaling pathway	8	CDK2 EGFR IGF1R IL2 PGF PIK3CG PRKCA VEGFA
3	MicroRNAs in cancer	6	EGFR MMP9 ABCB1 PRKCA PTGS2 VEGFA
4	Proteoglycans in cancer	6	EGFR IGF1R MMP9 PRKCA TNF VEGFA
6	* MAPK signaling pathway	6	EGFR IGF1R PGF PRKCA TNF VEGFA
5	Serotonergic synapse	5	CYP2D6 ALOX5 APP PRKCA PTGS2
8	Focal adhesion	5	EGFR IGF1R PGF PRKCA VEGFA
10	Transcriptional misregulation in cancer	5	CDK9 ELANE IGF1R MMP9 PPARG
11	Chemical carcinogenesis	5	CYP3A4 EGFR EPHX2 PRKCA VEGFA
13	Rap1 signaling pathway	5	EGFR IGF1R PGF PRKCA VEGFA
16	Human cytomegalovirus infection	5	EGFR PRKCA PTGS2 TNF VEGFA
17	Ras signaling pathway	5	EGFR IGF1R PGF PRKCA VEGFA
19	Coronavirus disease	5	EGFR F2 IL2 PRKCA TNF
24	Human papillomavirus infection	5	CDK2 EGFR PTGS2 TNF VEGFA
7	* EGFR tyrosine kinase inhibitor resistance	4	EGFR IGF1R PRKCA VEGFA
9	Bile secretion	4	CYP3A4 ABCB1 SLC5A1 ABCG2
12	Endocrine resistance	4	CYP2D6 EGFR IGF1R MMP9
14	Prostate cancer	4	CDK2 EGFR IGF1R MMP9
15	AGE-RAGE signaling pathway in diabetic complications	4	NOX4 PRKCA TNF VEGFA
18	HIF-1 signaling pathway	4	EGFR IGF1R PRKCA VEGFA

20	Relaxin signaling pathway	4	EGFR MMP9 PRKCA VEGFA
21	Phospholipase D signaling pathway	4	EGFR F2 PIK3CG PRKCA
22	Oxytocin signaling pathway	4	EGFR PIK3CG PRKCA PTGS2
23	Hepatitis B	4	CDK2 MMP9 PRKCA TNF

* Pathways involved in acute renal failure

Table S4. top cellular components enriched to the target genes identified as targets to identified compounds from JGF

cellular components ID	Description	Fold Enrichment	Enrichment FDR	nGenes	Pathway Genes
GO:0043235	Receptor complex	7.234681787	1.38E-07	15	422
GO:0045177	Apical part of cell	6.290286976	2.19E-06	14	453
GO:0048471	Perinuclear region of cytoplasm	4.419038497	1.09E-05	17	783
GO:0070062	Extracellular exosome	3.078543647	7.09E-08	35	2314
GO:1903561	Extracellular vesicle	3.048245614	7.09E-08	35	2337
GO:0043230	Extracellular organelle	3.045639162	7.09E-08	35	2339
GO:0065010	Extracellular membrane-bounded organelle	3.045639162	7.09E-08	35	2339
GO:0005615	Extracellular space	2.694698189	7.68E-09	47	3550
GO:0005615	Extracellular region	2.228493222	1.25E-07	51	4658

GO:0031982	Vesicle	2.222409983	4.39E-07	48	4396

Table S5. Top Molecular functions enriched to the target genes identified as targets to identified compounds from JGF

molecular function ID	Pathway	Fold Enrichment	Enrichment FDR	nGenes	Pathway Genes
GO:0004672	Protein kinase activity	7.741453	1.56E-12	24	631
GO:0016773	Phosphotransferase activity, alcohol group as acceptor	6.583365	2.27E-11	24	742
GO:0016491	Oxidoreductase activity	6.337639	5.62E-12	26	835
GO:0016301	Kinase activity	6.000463	4.29E-11	25	848
GO:0016772	Transferase activity, transferring phosphorus-containing groups	5.218864	1.92E-10	26	1014
GO:0043168	Anion binding	3.495842	1.56E-12	45	2620
GO:0000166	Nucleotide binding	3.419332	7.51E-11	40	2381
GO:0036094	Small molecule binding	3.41827	1.56E-12	46	2739
GO:1901265	Nucleoside phosphate binding	3.417896	7.51E-11	40	2382

GO:0140096	Catalytic activity, acting on a protein	3.230726	1.92E-10	41	2583

Table S6. Top Biological process enriched to the target genes identified as targets to identified compounds from JGF

Biological process ID	Description	Fold Enrichment	Enrichment FDR	nGenes	Pathway Genes
GO:0120254	Olefinic compound metabolic process	21.93798	1.37E-16	18	167
GO:1901701	Cellular response to oxygen-containing compound	5.735584	3.81E-16	37	1313
GO:1901700	Response to oxygen-containing compound	5.221713	1.44E-19	47	1832
GO:0006468	Protein phosphorylation	5.094458	8.04E-17	42	1678
GO:0042127	Regulation of cell population proliferation	4.704733	9.45E-16	42	1817
GO:0008283	Cell population proliferation	4.647818	4.39E-18	48	2102
GO:0016310	Phosphorylation	4.435647	9.45E-16	44	2019

GO:0070887	Cellular response to chemical stimulus	3.760986	5.78E-23	68	3680
GO:0010033	Response to organic substance	3.300579	8.04E-17	60	3700
GO:0065008	Regulation of biological quality	3.298254	2.44E-20	69	4258
